# Tensile and Shear Testing of Basalt Fiber Reinforced Polymer (BFRP) and Hybrid Basalt/Carbon Fiber Reinforced Polymer (HFRP) Bars

**DOI:** 10.3390/ma13245839

**Published:** 2020-12-21

**Authors:** Kostiantyn Protchenko, Fares Zayoud, Marek Urbański, Elżbieta Szmigiera

**Affiliations:** Faculty of Civil Engineering, Warsaw University of Technology, 00-637 Warsaw, Poland; fares.zayoud.stud@pw.edu.pl (F.Z.); m.urbanski@il.pw.edu.pl (M.U.); e.szmigiera@il.pw.edu.pl (E.S.)

**Keywords:** Fibers Reinforced Polymer (FRP), Hybrid Fibers Reinforced Polymer (HFRP), Basalt Fibers Reinforced Polymer (BFRP), tensile strength testing, shear strength testing

## Abstract

The use of sustainable materials is a challenging issue for the construction industry; thus, Fiber Reinforced Polymers (FRP) is of interest to civil and structural engineers for their lightweight and high-strength properties. The paper describes the results of tensile and shear strength testing of Basalt FRP (BFRP) and Hybrid FRP (HFRP) bars. The combination of carbon fibers and basalt fibers leads to a more cost-efficient alternative to Carbon FRP (CFRP) and a more sustainable alternative to BFRP. The bars were subjected to both tensile and shear strength testing in order to investigate their structural behavior and find a correlation between the results. The results of the tests done on BFRP and HFRP bars showed that the mechanical properties of BFRP bars were lower than for HFRP bars. The maximum tensile strength obtained for a BFRP bar with a diameter of 10 mm was equal to approximately 1150 MPa, whereas for HFRP bars with a diameter of 8 mm, it was higher, approximately 1280 MPa. Additionally, better results were obtained for HFRP bars during shear testing; the average maximum shear stress was equal to 214 MPa, which was approximately 22% higher than the average maximum shear stress obtained for BFRP bars. However, HFRP bars exhibited the lowest shear strain of 57% that of BFRP bars. This confirms the effectiveness of using HFRP bars as a replacement for less rigid BFRP bars. It is worth mentioning that after obtaining these results, shear testing can be performed instead of tensile testing for future studies, which is less complicated and takes less time to prepare than tensile testing.

## 1. Introduction

Concrete structures built during the previous century will be in need of modernization and renovation, which will lead to an increase in resources and costs associated with these buildings. Hence, it is necessary to develop inexpensive and efficient modernizing techniques in order to prevent structural damage. Throughout the years, researchers have experimented with different materials in various contexts, and Fiber Reinforced Polymers (FRP) have proved to be an applicable material for strengthening and reinforcing concrete structures.

For decades, FRP has been widely used in the construction industry as a substitute for steel due to its lightweight, increased corrosion resistance, and improved strength. FRP can be used in a wide range of applications in construction, such as reinforcement of concrete, formwork, modular structures, bridge decks, and external reinforcement for strengthening structures.

FRP reinforcement exhibits a linear elastic behavior prior to failure but fails in a brittle manner. The weakness of FRP reinforcement in terms of its mechanical behavior is observed in the transverse direction, which can be seen in shear resistance testing, where the strength characteristics of tested samples are significantly reduced. Moreover, the FRP reinforcement lacks plastic behavior. However, FRP reinforcement has several advantages, such as high tensile strength in the longitudinal direction, long durability, high corrosion resistance, and easy application.

Tensile tests have been conducted by several researchers over the past decades on different FRP bars to determine their mechanical and physical properties [1,2,3,4,5,6,7]. Conclusions drawn from these earlier studies [4,5,6,7,8,9,10] indicate that the mechanical characteristics of FRP bars are mainly dependent on fiber volume and type. Studies have also shown that FRP bars exhibit elastic linear behavior up to failure, with a modulus of elasticity lower than that of steel.

In the studies of Walsh, Chang, and Wallenberger et al. [8,9,10], extensive research can be found on the most common fibers used to manufacture FRP rebars, such as carbon, aramid, and glass. Hollaway et al. [11] found that fibers exhibited a linear elastic behavior under tensile loading up to failure without showing any yield.

Nanni et al. [12] investigated the tensile properties of hybrid rods for concrete reinforcement. The tested specimens were made of 1.0 m long rods, including the anchorage system. The results of uniaxial tensile testing, conducted in order to determine stress–strain relationships and tensile properties, showed that the stress–strain diagrams displayed a bilinear behavior.

Zhishen Wu et al. found that the tensile modulus of the fibers influenced the fatigue failure mode, where the Glass Fiber Reinforced Polymers (GFRP) and Basalt Fiber Reinforced Polymers (BFRP) composites exhibited transverse cracks that indicated a stress transfer to the adhesive layer with a modulus of about 90 GPa, whereas the Carbon Fiber Reinforced Polymers (CFRP) and Poly(p-phenylene benzobisoxazole) (PBO) composites showed longitudinal cracks along the test coupons with a modulus of approximately 250 GPa [13].

Marta Kosior-Kazberuk performed an investigation on the application of Basalt FRP (BFRP) bars and Hybrid FRP (HFRP) bars as reinforcement for full-scale concrete beams. The results showed that the FRP bars used in the tensile zone worked in a similar way to steel reinforcement. Rapid destruction in the tensile zone with crushing of concrete in the compression zone, deformation of compressed bars, stirrups breaking within cracks, and failure of tensile reinforcement were observed [14].

Shi et al. [15] investigated the tensile behavior of FRP and Hybrid FRP sheets in Freeze–Thaw (FT) cycling environments. Additional degradation of the tensile strength and rupture elongation of FRP sheets were observed as a result of sustained loading during FT cycling, while the elastic modulus of the FRP sheets was not influenced by the sustained loading during FT cycling. Hence, it is necessary to consider service loads during durability tests to reflect the actual conditions faced by FRP composites in harsh civil engineering environments.

Many researchers have studied the shear strength behavior of different FRP bars. The limited application of BFRP in the past decades led to the limited investigation of its shear behavior. However, Benmokrane et al. [16] proved that the type of the resin plays a significant role in the inter-laminar shear strength of BFRP bars, as BFRP bars with epoxy resin showed higher durability than BFRP bars with vinyl ester resin. In addition, the durability of GFRP bars was lower than for BFRP bars with epoxy resin.

Alam et al. [17] studied the shear strength of FRP reinforced members without transverse reinforcement and reported that the normalized shear strength increased linearly with the cube root of the axial stiffness of the reinforcing bars. However, there were similar results for the shear strength of FRP reinforced elements where it was proportional to the axial stiffness of the longitudinal reinforcement obtained by Alam et al. [17], El-Sayed et al. [18,19], and Razaqpur et al. [20]. Hence, the conclusion that the shear strength was proportional to the amount of longitudinal reinforcement was reached by Alkhrdaji et al. [21], and the effect of the reinforcement stiffness and amount of reinforcement on the shear strength of FRP elements, despite no significant influence of the longitudinal reinforcement ratio on the shear strength, was observed by Yost et al. [22].

Khalifa et al. [23], Täljsten [24], and Triantafillou [25] developed a theory that aims at describing FRP stress distribution along a shear crack with closed-form equations, as opposed to the regression-based formula that Triantafillou et al. [26] introduced. FRP contribution to the resisting shear and the FRP resultant across the crack can be computed when this formula is correctly defined.

Based on the literature, axial tensile testing is most commonly used for defining the mechanical properties of composite bars, and some of the researchers have studied the shear properties of the bars through tensile testing. Current research is concentrated on the defining properties of BFRP and HFRP bars through axial-tensile testing and shear testing, as well as finding a correlation between these two testing methods. 

The variables in this study were types of FRP bars and the diameter of bars. The outcomes were compared, and a correlation between the results was found. Establishing this correlation allowed the identification of mechanical properties of FRP bars basing on shear testing, which is easier to prepare and takes less time than tensile testing. 

### Purpose and Novelty of the Work

RC bending elements are the basic structural components that cover the operational load as well as the load from partition walls and transfer them further to vertical elements (such as columns and load-bearing walls). Reinforcement of these elements should be characterized by high strength values and appropriate corrosion resistance. In order to determine these properties, strength tests should be carried out. On the one hand, the tensile strength test for FRP bars is complex, relatively labor-intensive, and expensive. On the other hand, testing the shear strength of FRP bars is simple, low-cost, and fast. By carrying out a quick and simple shear test, it is possible to determine the tensile strength and stiffness (modulus of elasticity) of both homogeneous BFRP bars and HFRP bars on the basis of the method presented in the article. Additionally, the method allows optimizing the tensile strength and stiffness based on the determined correlation between shear strength and tensile strength.

## 2. The Concept of Hybrid FRP Bars

In spite of the fact that FRP has numerous prevalent material properties, such as high corrosion resistance, high specific stiffness, high specific strength, and durability, the brittle nature and high cost of FRP limits its extensive usage in many industries. To overcome these obstacles, combinations of FRP and ordinary materials are being explored by several researchers [27]. Appropriate properties can be obtained by utilizing a combination of FRP and steel [28,29] or by combining different sorts of FRP materials in one structure [30]. The advantages of hybrid structural systems include cost-effectiveness and the ability to optimize the cross-section based on the material properties of each constituent material [31].

HFRP bars can be an optimal solution since hybrid bars have a better combination of mechanical properties and are less expensive than FRP bars with homogeneous fibers. On the other hand, they are corrosion resistant and have significantly better strength characteristics than conventional steel reinforcements [32]. Hybridization between different constituents focuses on picking out the advantages of each constituent, while the disadvantages can be improved [33]. 

Due to the high cost of carbon, the ideal solution is the usage of HFRP bars, where the part of low-cost basalt fibers was substituted by high-cost carbon fibers. Hence, the hybridization of carbon/basalt fibers is less expensive than CFRP; concurrently, this combination is characterized by better mechanical properties in comparison with BFRP [34,35,36].

Carbon fibers are chosen due to their high properties in the longitudinal direction as well as their strong anisotropy. However, basalt fibers were chosen for their environmentally friendly producing process and low-cost. In addition, basalt fibers are significantly less brittle when used in composites [37]. An additional reason for such selection was similar strain parameters for both types of fibers. Mechanical properties of the constituents utilized for preparing HFRP bars are represented in Table 1.

**Figure 1:**
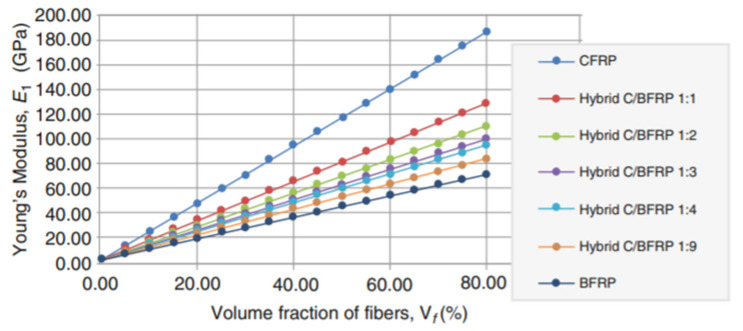
Theoretical relationship of fiber volume fraction and Young′s modulus (obtained from Voigt’s Model) for Hybrid Carbon/Basalt FRP [36,37].

Composite materials properties can be calculated according to the Rule of Mixtures (ROM) (axial loading–Voigt model) based on the literature [34,35,36]. Based on the Rule of Mixtures (ROM) equation, Young’s modulus and other parameters were determined for the Hybrid Carbon/Basalt FRP (HC/BFRP) and for different combinations depending on carbon to basalt fiber volume fractions. Different levels of fibers substitution (carbon-to-basalt C/B) were proposed: 1:1, 1:2, 1:3, 1:4, and 1:9, which is represented in Figure 1 [36].

The Voigt model does not consider the location of the fibers; therefore, numerical analysis was made for two configurations: (i) carbon fibers in the core region and basalt fibers in the near-surface region, (ii) carbon fibers in the near-surface region and basalt fibers in the core region of the bar section. The numerical simulation of the tensile strength test for HFRP bars was performed by Finite Element Methods (FEM). The bars consisted of two parts, the core region (cylindrical form) and the surface region (tube form), which were perfectly interconnected. The constant pressure was applied on both sides along the longitudinal axis. The obtained results from numerical modeling were compared with analytical considerations and were found to be in a good convergence with each other.

Numerical results indicated that the arrangement of the fibers was not of high importance for the final mechanical properties of HFRP bars with different combinations of fibers. More on the analytical and numerical investigation of Hybrid FRP bars can be found in companion papers [36,37]. Two different HFRP bars’ configurations were produced by the manufacturing company. For the first configuration, carbon fibers were placed in the core region, while the second configuration was when carbon fibers were in the near-surface region. However, some technological issues were observed while placing carbon near-surface, increasing heterogeneity in fiber distribution, and local scorching of carbon fibers caused by rising temperature. Finally, carbon fibers were placed mostly in the core region of the bar. 

HFRP bars utilized for this work were composed of epoxy matrix and basalt fibers, with a volume ratio of 1:4 (i.e., 16% carbon fibers, 64% basalt fibers, and 20% matrix). Due to this substitution, HFRP bars are characterized by a much higher stiffness, which enables more efficient application as reinforcement for concrete elements subjected to bending and compression [37,38,39]. More about the hybridization of FRP bars used in work can be found in these companion papers [38,39].

In the tensile and shear strength tests conducted, two types of FRP were used: BFRP and HFRP. Twisting FRP braids were done in order to improve adhesion with concrete (the equivalent of ribbing for steel bars) [36].

## 3. Tensile Strength Test

### 3.1. Experimental Procedure

The tensile strength testing of different BFRP and HFRP bars was carried out according to the ACI 440.3R and ASTM D7205/D7205M methodologies [40].

In the tensile strength test, anisotropic FRP materials of different diameters were tested according to the methodologies. The two different types of FRP bars had different diameters, specifically Ø6, Ø8, Ø10, Ø12, Ø14, and Ø18 mm. Hence, for each type of BFRP and HFRP bar, five samples of each diameter were tested. In total, tensile tests were carried out on sixty bars.

Basalt fiber strength in the transverse direction is very low compared to its extremely high longitudinal strength. Hence, the use of appropriate anchorage at both ends of the bar subjected to the tensile test on the testing apparatus is a requirement. To meet this requirement, two steel pipes with a length of 400 mm, an external diameter of 40 mm, and a thickness of 5 mm each were designed. The steel caps at the end of the steel pipes were designed with an opening in the center for the bar.

**Figure 2:**
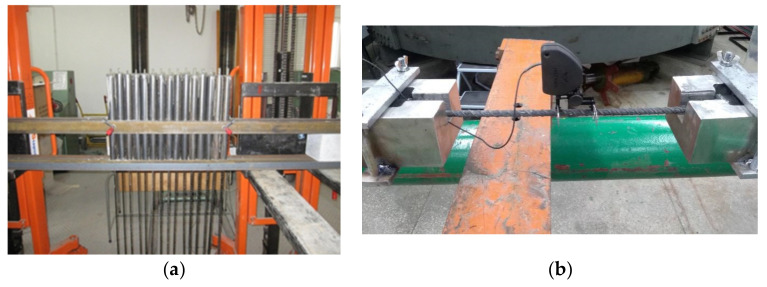
(**a**) Basalt Fiber Reinforced Polymers (BFRP) bars prepared for testing, (**b**) Hybrid Fiber Reinforced Polymers (HFRP) bars in the tensile testing machine.

The free space between the bar and the pipe was filled with a special adhesive layer. The material filling the anchoring pipes was determined on the basis of previous tests done at the Warsaw University of Technology [39,40]. Figure 2a shows the BFRP bars prepared for tensile strength testing; Figure 2b shows the HFRP bars in the tensile testing machine.

The tensile strength test was carried out in accordance with [40] standard for pultruded FRP bars. However, the tensile strength of the specimens can be calculated by dividing the measured load by the transverse cross-sectional area of FRP bars of the corresponding type, *A_i,FRP_*. To experimentally determine the modulus of elasticity of FRP bars, Equation (1) can be used.

(1) $$E_{11,iFRP}=\frac{\left(P_{1}-P_{2}\right)}{\left(\varepsilon_{1}-\varepsilon_{2}\right)\cdot A_{i,FRP}}$$

where:

- $E_{11,iFRP}$ —modulus of elasticity of corresponding FRP bars along the longitudinal axis,
- $P_{1}$ and $P_{2}$—applied loads corresponding to 50% and 25% of the ultimate load, respectively,
- $\varepsilon_{1}$ and $\varepsilon_{2}$—strains corresponding to 50% and 25% of the ultimate strains, respectively, *A_i,FRP_*—cross-sectional area for BFRP and HFRP bars, respectively.

### 3.2. Calculations and Results

The deformation measurement was performed up to 70% of the breaking load of the bars due to the possibility of strain gauge damage.

The average values obtained for BFRP and HFRP bars of tensile strength, tensile strain, and tensile modulus for six different diameters of each type are presented in Table 2, Table 3 and Table 4, respectively.

**Figure 3:**
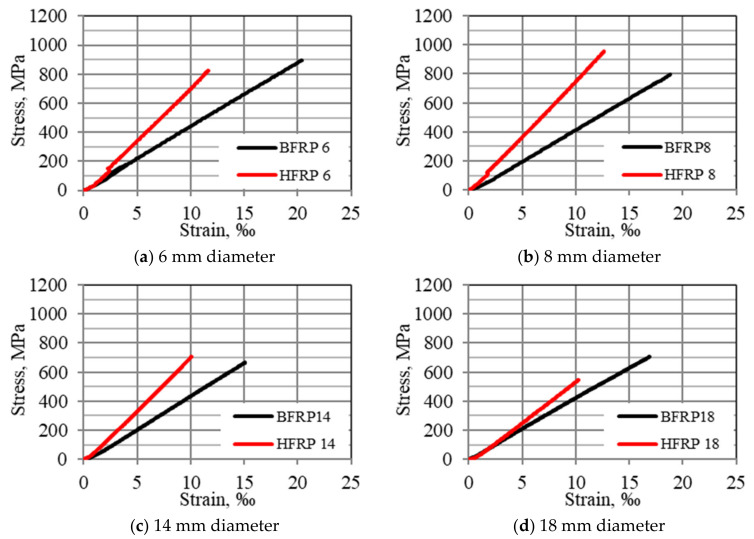
Stress–strain relationships of tension Fiber Reinforced Polymers (FRP) bars with mean values for diameters: (**a**) Ø6, (**b**) Ø8, (**c**) Ø14, (**d**) Ø18 mm.

Figure 3 displays the stress–strain relationships of tension FRP bars tested with mean values for diameters Ø6, Ø8, Ø14, and Ø18 mm.

In the tensile property values obtained for BFRP and HFRP, the deformation values obtained for HFRP were lower than the analogous deformation values for BFRP at rupture.

**Figure 4:**
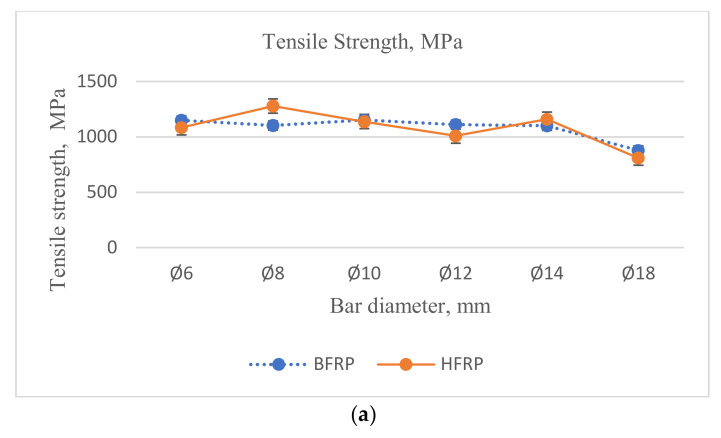

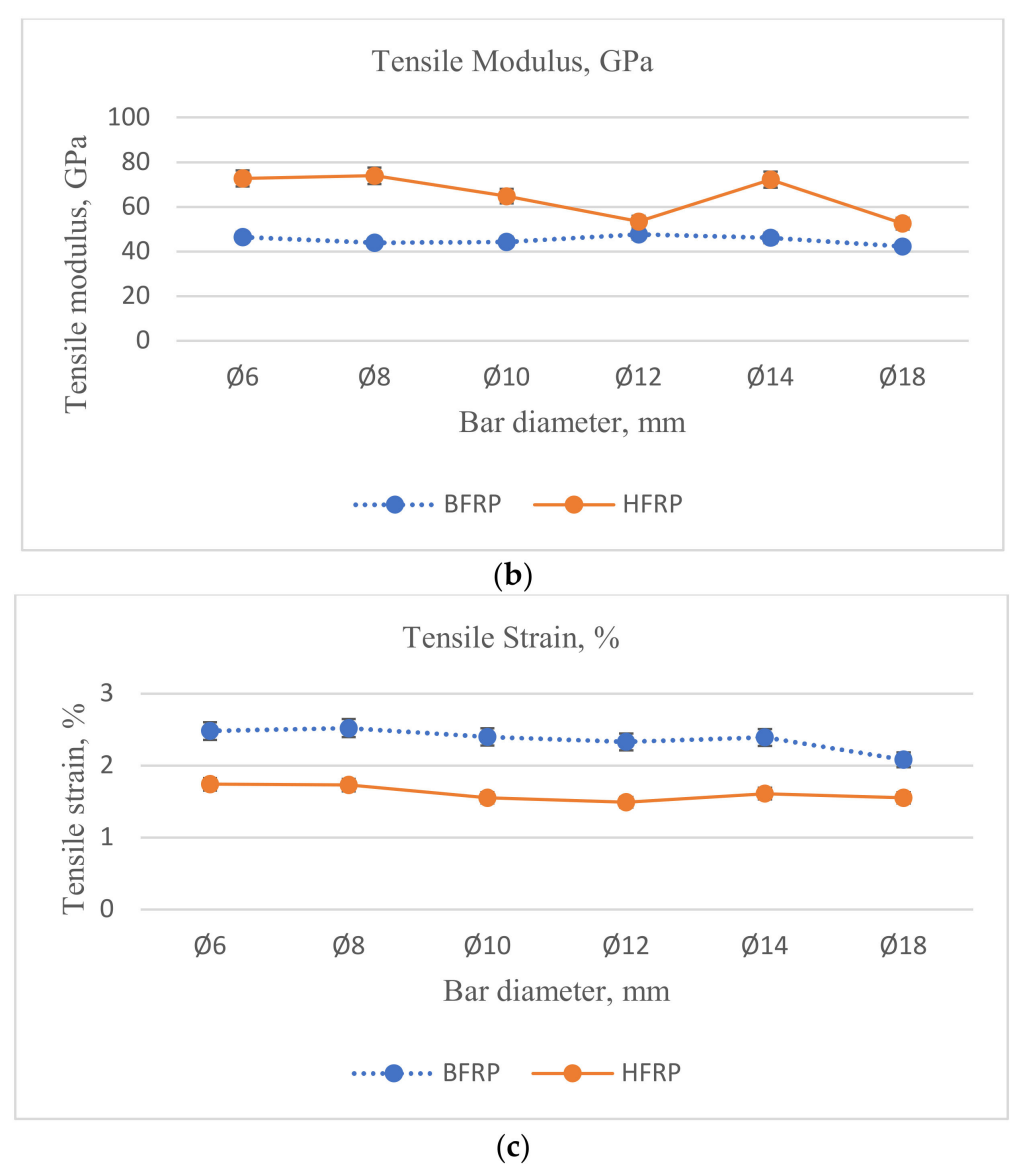
Mechanical properties of FRP bars obtained from tensile testing: (**a**) Tensile strength, (**b**) Modulus of elasticity, (**c**) Tensile strain.

Additionally, comparing the (Coefficient of Variation, COV) values of the two types of FRPs used in the test, much higher (COV) values were obtained for HFRP bars compared to the tested BFRP bars. However, this phenomenon might occur due to technical issues indicated in the previous chapter, which might occur during the manufacturing of bars composed of several FRP roving. Hence, the (COV) value depends on the diameter of the bars. The average (COV) values for BFRP bars were twice as small as the corresponding COV values for HFRP. The changes in mechanical properties for the different diameters and FRP types are represented in Figure 4.

Comparing the tensile strength of BFRP and HFRP bars with diameters of Ø8 and Ø14 mm, the values for BFRP bars of diameter Ø8 mm were smaller than HFRP bars of the same diameter by 15% and slightly smaller for bars of diameter Ø14 mm by approximately 5%. However, the tensile strength values for BFRP bars with diameters Ø6, Ø10, Ø12, and Ø18 mm were slightly higher than HFRP bars with the same diameters. Values for BFRP bars with diameters Ø6, Ø12, and Ø18 mm were slightly higher than HFRP bars by approximately 5%, and for diameter Ø10 mm by approximately 2%.

The rupture strain for all HFRP bar diameters is about 1/3 lower than the rupture strain of the BFRP bars. This is due to the smaller ultimate tensile strains of the carbon fiber (compared to the strain of basalt fiber) contained in the HFRP bars.

Observations made during the study confirm theoretical forecasts. The ultimate strain of hybrid bars corresponds to the strain of its fibers, which are characterized by the lowest ultimate strain among the fibers contained in the HFRP bar. The values obtained for the tensile modulus of elasticity for HFRP bars for all diameters were greater than the values obtained for the tensile modulus of elasticity for BFRP bars for all diameters. 

The results obtained during this test confirm the viability of using HFRP bars as a replacement for less rigid BFRP bars.

## 4. Shear Strength Testing

### 4.1. Experimental Procedure

The shear strength test of FRP bars was carried out in accordance with the methodology described in ACI 440.3R-04 and ASTM D7617/D7617M–11 [40]. 

**Figure 5:**
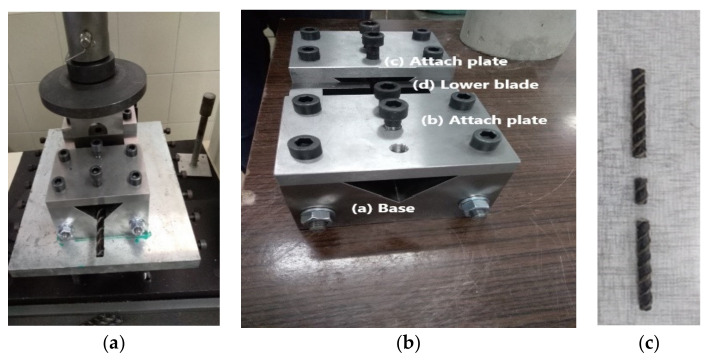
(**a**) Double shear testing device, Instron 3382, (**b**) Description of Instron 3382 testing machine, (**c**) Shear failure of the FRP bar.

The shear strength testing was carried out on BFRP and HFRP bars with diameters Ø6, Ø8, Ø10, Ø12, Ø14, and Ø18 mm. For the shear strength test, in order to ensure that the bar sample shears in two planes simultaneously, a steel device was constructed with blades converging along a surface perpendicular to the sample axis. The device consists of one upper blade, two lower blades, and a sample holder. The sample holder has a V-shaped groove for placing FRP samples and a rectangular notch for holding the lower and upper blades in the center of the upper part of the device. The sample holder has 100 mm width, 110 mm height, and 230 mm length. It is attached to the instrument stand with screws that stabilize it in order to eliminate horizontal movement of the bar. Figure 5 represents the components of the shear testing device and the shear failure of the FRP bar. A total of 60 specimens of BFRP and sixty specimens of HFRP bar were tested, where five test specimens were produced for each diameter of every FRP bar type used during the shear strength test.

All FRP bars that were tested for shear strength were composed of a spiral braid of basalt fiber. All the FRP bars were tested at speeds up to 50 MPa/min for a range of time between 3.5 min and 4.5 min, with the test ending when a sharp decrease in shear stress was observed.

According to ACI 440.6-08 and ASTM A615/A615M [40] methodologies, shear stress fs was calculated according to Equation (2) using an equivalent cross-section of bars.

(2) $$f_{s}=\frac{F_{u}}{2\cdot A_{i,FRP}}$$

where:

- $F_{u}$ —the maximum shear force, N.
- $A_{i,FRP}$ —the equivalent cross-section of the tested bar, mm^2^.

### 4.2. Calculations and Results

**Figure 6:**
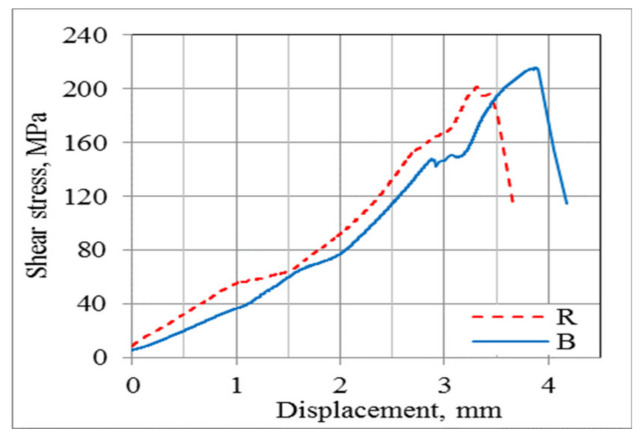
Stress-displacement diagram of transverse shear test for two HFRP bars with a mean of 14 mm.

Failure of the tested bars due to shearing could occur in two ways; two failure modes are shown in the Figure 6. The blue “B” line (straight line), which occurred most often, was typical for simultaneous shearing of a bar in two planes. In contrast, the red “R” (dot line) graph showed the situation when shearing occurred in one and then slightly later in the other cross-section of the tested bar. 

**Figure 7:**
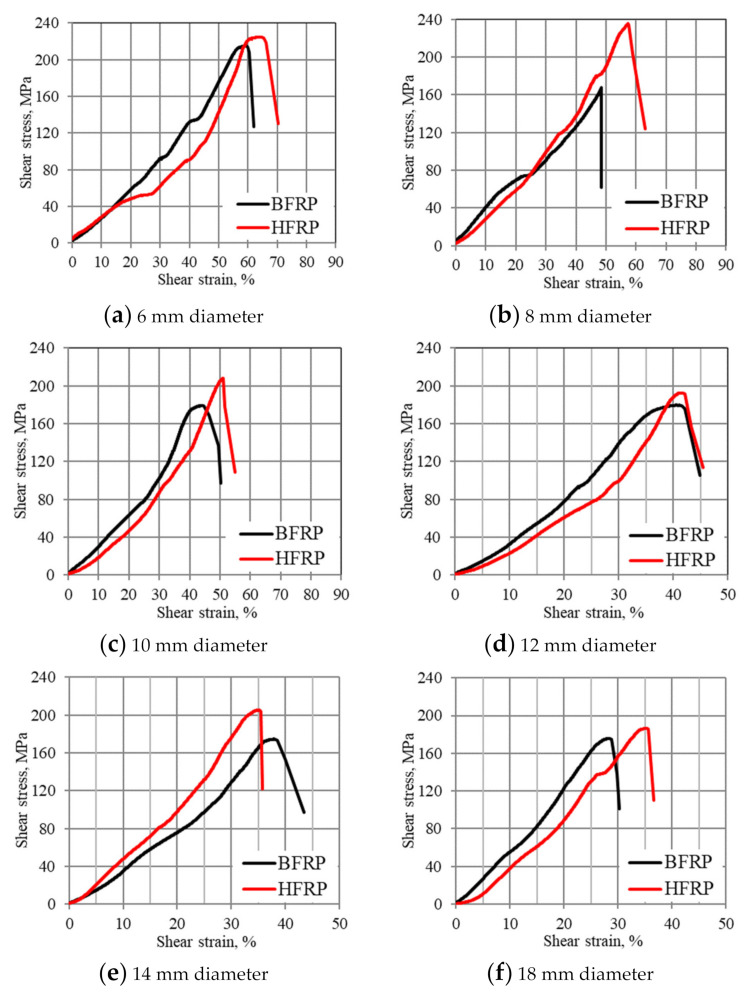
Shear stress–strain relationship of FRP bars tested with means. (**a**) Ø6, (**b**) Ø8, (**c**) Ø10, (**d**) Ø12, (**e**) Ø14, (**f**) Ø18 mm.

Figure 7 shows a relationship of shear stress to strain for three types of BFRP and HFRP bars with nominal diameters of 6, 8, 10, 12, 14, 18 mm.

It was impossible to clearly determine the values of the shear modulus due to the non-uniform slope of the shear curves, in contrast to the stress–strain relationship. Comparing BFRP bars to HFRP bars, it was noticed that BFRP bars had greater shear deformation and less shear strength compared to HFRP bars. Table 5 shows shear strength values obtained for different FRP bars.

The type of FRP bar affects the COV variation for shear strength. The quite significant spread of FRP bar test results confirms that they are heterogeneous materials consisting of a matrix and fibers. Studies show that the average lateral shear strength of BFRP bars ranges from 170 to 210 MPa. While for HFRP bars, it is up to 20% higher due to the participation of carbon fibers.

**Figure 8:**
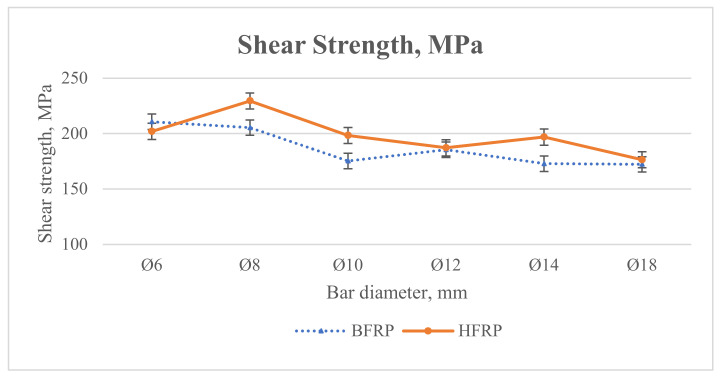
Comparison of average shear strength testing results of different FRP bars for all diameters.

The transverse shear strength displays a slight downward trend as the bar diameter increases, most evidently in HFRP bars. However, minor variation is observed in the shear strength data, with the COV greater than in the rebar tension tests. Figure 8 shows the comparison of shear strength of FRP bars for different diameters.

Comparing the average shear strength of BFRP bars to HFRP bars, the values for BFRP bars were lower than the average shear strength values of HFRP bars except for diameter Ø6 mm, where it was slightly higher by approximately 1%. However, regarding the other diameters, the average shear strength of BFRP bars was lower for diameters Ø8, Ø10, and Ø14 mm by approximately 19% and for diameters Ø12 and Ø18 mm by approximately 1%. This might suggest that the shear strength of BFRP bars was lower than for HFRP.

## 5. Results and Discussions

From the results obtained from the tensile and shear strength testing of BFRP and HFRP with different diameters, a correlation was made between the obtained values in order to find a method for calculating tensile strength from the data obtained from shear strength experimental testing.

In Table 6, the correlation between shear strength and tensile strength of different types of FRP bars with different diameters is shown.

From Table 6, the average ratio (*k_d_*) according to bar diameter can be calculated from Equation (3):

(3) $$k_{d}=\frac{\sum \frac{f_{s}}{f_{t}}}{n_{t}}$$

where $n_{t}$–number of tested bar types ($n_{t}$ = 2).

Based on Equation (3), the coefficient of accuracy corresponding to a given diameter can be identified. The values are presented in Table 7. Table 8 and Table 9 represent comparison between the tensile strength values of experimental and calculated results.

Accuracy represented in Table 9 was calculated according to Equation (4):

(4) $$FRP_{accuracy}=\frac{f_{t}\left(exp\right)-f_{t}\left(\emptyset \right)}{f_{t}\left(exp\right)}\;\cdot \;100$$

where:

- $f_{t}\left(exp\right)$ —Tensile strength obtained experimentally,
- $f_{t}\left(\emptyset \right)$ —Tensile strength obtained theoretically.

**Figure 9:**
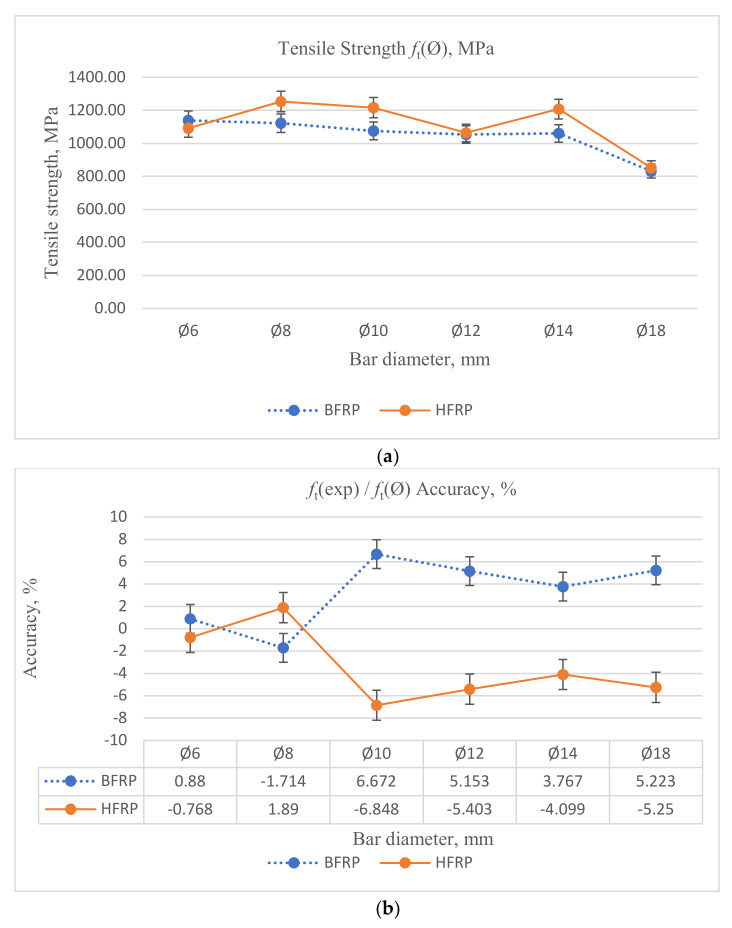
(**a**) Tensile strength obtained theoretically. (**b**) Accuracy according to coefficient (*k_d_*).

Figure 9 presents results obtained from Equation (3) according to the diameter of FRP bars proposed in order to calculate the tensile strength of FRP bars from the shear strength obtained experimentally; the accuracy of the values obtained theoretically using coefficient (*k_d_*) varied between −7% and +7% in comparison with the tensile strength experimental values, using the Equation (5) for BFRP and HFRP:

(5) $$f_{tensile}=\frac{f_{shear}}{k_{d}}$$

Thus, it is possible to obtain the tensile strength of FRP bars by applying shear test strength only using Equation (5), taking into consideration the additional number of samples to be tested in order to eliminate errors and obtaining better accuracies. The coefficient (*k_d_*) can be identified for any specific type of FRP, which will allow us to determine tensile strength based on shear testing.

The modulus of elasticity for the tested bar diameters depends mostly on the volume fraction of carbon and basalt fibers. The differences in volume fraction can be caused due to a various number of rovings used in the pultrusion process, which do not correspond to the bar diameter. In the case of BFRP bars with diameters of 10, 12, and 14 mm, the volume fraction of basalt fibers was greater than the average value by about 10%, while for the diameters of 18 mm, it was lower by over 10%. On the other hand, for the HFRP bars with diameters of 14 mm, the volume fraction of fibers was higher by more than 10% than the average value, while for the diameters of 18 mm, it was 20% lower.

The tensile strength, in turn, is influenced by the shear lag effect, which causes that as the diameter of the bar increases, there is an increasing disproportion of the stress of the fibers in the cross-section of the bar.

Summarizing, the values of modulus of elasticity and tensile strength, as well as shear strength, depend on a combination of two factors, specifically the volume fraction of fibers and the shear lag effect.

## 6. Conclusions

The tests conducted in this study provided insight into the mechanical behavior of the types of FRP bars analyzed. Considering the results observed during tests done on BFRP bars for all diameters, it can be seen that the modulus of elasticity was quite low compared to HFRP bars, lower, on average, by approximately 18%. However, the main objective of this research was to check the maximum strength of HFRP and how it is compared to BFRP, as well as to find the stress–strain correlation. The influence of material hybridization was explored in order to examine the possibility of using less complicated shear testing as an alternative to tensile testing.

Based on experimental tests and numerical and analytical considerations for BFRP and HFRP bars, the following conclusive remarks can be drawn:

1. The tensile stress–strain correlation of BFRP bars was tested for given diameters. The average maximum stress obtained equaled to approximately 700 MPa, with an average strain approximately equal to 17%. However, the HFRP bars tested exhibited average maximum stress approximately equal to 725 MPa with the average strain approximately equal to 11%. This confirmed the viability of using HFRP bars as a replacement for less rigid BFRP bars.
2. Based on the tests performed, HFRP bars exhibited better results in terms of their tensile stress–strain relationship. However, comparing the obtained results for HFRP to BFRP bars, the tensile strength and modulus of elasticity of HFRP bars were higher by approximately 68% and 16%, respectively. The 6% lower elongation of HFRP bars could be explained by the superior extensibility properties of carbon fiber.
3. Additionally, according to the shear stress–strain relationship, better results were obtained for HFRP bars with average maximum shear stress equal to 214 MPa, which was approximately 22% higher than the average maximum shear stress obtained for BFRP bars. However, HFRP bars obtained a lower shear strain, 57% compared to BFRP bars.
4. Comparing experimental to analytical/numerical calculations done by the authors, the predicted analytical/numerical results were much better than those obtained experimentally. The difference in mechanical properties of FRP bars could be explained by the exact chemistry of the polymer matrix and the strength of polymer/BFRP or polymer/HFRP interactions.
5. Comparing the tensile stress–strain relationship to shear stress–strain for all bars and diameters, it was observed that BFRP and HFRP bars could withstand more tensile force and had a lower accompanying strain in tensile tests than shear tests.

The preparation for shear testing is less complicated than for tensile testing; therefore, on the basis of the results and observations, shear testing can substitute tensile testing for this class of composite materials.

## Figures and Tables

**Table 1:** Properties of constituents utilized for preparing Hybrid Fiber Reinforced Polymers (HFRP) rebars [34].

Properties	Units	Carbon Fibers LS	Basalt Fibers	Epoxy Resin
Density	g/cm^3^	1.90–2.10	2.60–2.80	1.16
Diameter	μm	7.00–11.00	11.20–13.40	–
*E*_11_	GPa	232.00	89.00	3.45
*E*_22_	GPa	15.00	89.00	3.45
*v*_12_	–	0.28	0.26	0.35
*v*_23_	–	0.49	0.26	0.35
*G*_12_	GPa	24.00	21.70	1.28
*G*_23_	GPa	5.03	21.70	1.28
*σ*_11_	MPa	2500–3500	1153–2100	55–130

*E_ii_* is the modulus of elasticity along axis *i, v_ij_* is the Poisson ratio that corresponds to a contraction in direction *j* when an extension is applied in direction *i*, *G_ij_* is the shear modulus in direction *j* on the plane whose normal is in direction *i,* and *σ_ii_* is the tensile strength in the direction *i* [34].

**Table 2:** Tensile strength results of FRP bars.

Bar	Parameter	Ø6	Ø8	Ø10	Ø12	Ø14	Ø18
BFRP	*f_t_*, MPa	1148.81	1103.33	1152.54	1111.00	1101.94	877.98
	SD *, MPa	41.18	22.87	35.27	40.23	28.54	11.17
	COV **, %	3.58	2.07	3.06	3.62	2.59	1.27
HFRP	*f_t_*, MPa	1083.78	1277.92	1138.65	1008.52	1160.06	809.44
	SD *, MPa	59.64	55.41	34.89	36.51	44.75	19.03
	COV **, %	4.72	4.34	3.06	3.62	3.86	2.35

* SD–Standard Deviation; ** COV–Coefficient of Variation.

**Table 3:** Modulus of elasticity of FRP bars.

Bar	Parameter	Ø6	Ø8	Ø10	Ø12	Ø14	Ø18
BFRP	*E_f_*, GPa	46.47	43.87	44.27	47.63	46.02	42.20
	SD, GPa	2.58	0.86	1.71	0.89	0.23	1.58
	COV, %	5.55	1.95	3.56	1.87	0.49	3.74
HFRP	*E_f_*, GPa	72.71	73.89	64.77	53.35	72.12	52.43
	SD, GPa	1.67	3.07	3.50	3.93	2.20	2.46
	COV, %	2.29	4.15	4.76	5.78	3.05	4.70

**Table 4:** Strains at FRP bars rupture.

Bar	Parameter	Ø6	Ø8	Ø10	Ø12	Ø14	Ø18
BFRP	*ε_t_*, %	2.48	2.52	2.40	2.33	2.39	2.08
	SD, GPa	0.15	0.05	0.15	0.11	0.06	0.10
	COV, %	5.92	2.09	6.09	4.60	2.71	4.84
HFRP	*ε_t_*, %	1.74	1.73	1.55	1.49	1.61	1.55
	SD, GPa	0.08	0.07	0.10	0.10	0.06	0.04
	COV, %	4.38	4.33	6.65	6.70	3.73	2.59

**Table 5:** Shear strength testing results of FRP bars for different diameters.

Bar	Parameter	Unit	Ø6	Ø8	Ø10	Ø12	Ø14	Ø18
BFRP	*f_s_*	MPa	210.66	205.37	175.33	185.46	172.85	172.25
	SD	MPa	3.84	11.57	6.46	4.70	8.59	6.90
	COV	%	1.82	5.63	3.68	2.54	4.97	4.01
HFRP	*f_s_*	MPa	202.04	229.44	198.31	187.09	196.84	176.35
	SD	MPa	17.51	4.06	6.17	5.99	12.06	9.75
	COV	%	8.67	1.77	3.11	3.20	6.13	5.53

**Table 6:** Shear strength to tensile strength ratio.

Bar Type	Parameter	Ø6	Ø8	Ø10	Ø12	Ø14	Ø18
BFRP	*f*_s_ / *f*_t_	0.183	0.186	0.152	0.167	0.157	0.196
HFRP	*f*_s_ / *f*_t_	0.186	0.180	0.174	0.186	0.170	0.218

**Table 7:** Coefficient of accuracy (*k_d_*) of FRP bars according to diameter.

FRP Bars	Ø6	Ø8	Ø10	Ø12	Ø14	Ø18
Coefficient of Accuracy, *k_d_*	0.185	0.183	0.163	0.176	0.163	0.207

**Table 8:** Tensile strength calculation according to diameter ratios (*f*_t_ = *f*_s_/*k_d_*).

Bar Diameter	*k_d_*	BFRP	HFRP
mm	–	MPa	MPa
Ø6	0.185	1138.70	1092.11
Ø8	0.183	1122.24	1253.77
Ø12	0.163	1075.64	1216.63
Ø14	0.176	1053.75	1063.01
Ø16	0.163	1060.43	1207.61
Ø18	0.207	832.12	851.93

**Table 9:** Comparison between the tensile strength values of experimental and calculated results.

Bar Type	Parameter	Units	Ø6	Ø8	Ø10	Ø12	Ø14	Ø18
BFRP	*f*_t_(exp)	MPa	1148.81	1103.33	1152.54	1111.00	1101.94	877.98
	*f*_t_(Ø)	MPa	1138.70	1122.24	1075.64	1053.75	1060.43	832.12
HFRP	*f*_t_(exp)	MPa	1083.78	1277.92	1138.65	1008.52	1160.06	809.44
	*f*_t_(Ø)	MPa	1092.11	1253.77	1216.63	1063.01	1207.61	851.93
BFRP Accuracy	*f*_t_(ex)/*f*_t_(Ø)	%	0.88%	−1.71%	6.67%	5.15%	3.77%	5.22%
HFRP Accuracy	*f*_t_(ex)/*f*_t_(Ø)	%	−0.77%	1.89%	−6.85%	−5.40%	−4.10%	−5.25%
